# Supplementary material for: A SoxB gene acts as an anterior gap gene and regulates posterior segment addition in a spider
Source: eLife. 2018 Aug 21;7:e37567. doi: 10.7554/eLife.37567 (PMC6167052; doi:10.7554/eLife.37567)
Supplement: Supplementary file 1. [file elife-37567-supp1.docx]

| **Fragment 1** | | | | | |
| --- | --- | --- | --- | --- | --- |
|  | **Wild Type** | **Class I** | **Class II** | **Class III** | **Dead** |
| **Cocoon 1** | 205 |  |  |  | 5 |
| **Cocoon 2** | 0 | 21 | 47 | 110 | 32 |
| **Cocoon 3** | 0 | 35 | 54 | 121 |  |
| **Cocoon 4** | 0 | 42 | 105 | 46 | 17 |
| **Cocoon 5** | 27 | 19 | 41 | 63 | 39 |
|  |  |  |  |  |  |
| **Fragment 2** | | | | | |
|  | **Wild Type** | **Class I** | **Class II** | **Class III** | **Dead** |
| **Cocoon 1** | 210 |  |  |  |  |
| **Cocoon 2** | 0 | 20 | 20 | 91 | 79 |
| **Cocoon 3** | 0 | 49 | 37 | 124 |  |
| **Cocoon 4** | 0 | 20 | 27 | 133 | 30 |
| **Cocoon 5** | 16 | 8 | 67 | 84 | 35 |
|  |  |  |  |  |  |
| **GFP dsRNA** | | | | | |
|  | **Wild Type** | **Dead** |  |  |  |
| **Cocoon 1** | 56 | 4 |  |  |  |
| **Cocoon 2** | 53 | 7 |  |  |  |
| **Cocoon 3** | 50 | 10 |  |  |  |
| **Cocoon 4** | 56 | 4 |  |  |  |
| **Cocoon 5** | 48 | 12 |  |  |  |
