# Supplementary material for: A SoxB gene acts as an anterior gap gene and regulates posterior segment addition in a spider
Source: eLife. 2018 Aug 21;7:e37567. doi: 10.7554/eLife.37567 (PMC6167052; doi:10.7554/eLife.37567)
Supplement: Supplementary file 2. [file elife-37567-supp2.docx]

| **Figure 3** | | | |
| --- | --- | --- | --- |
| **Gene/Protein** | **Sample Size** | **Class Used** | **Stage** |
| *Pt-dpp* | 30/30 | I and II pooled | 5 |
| *Pt-Ets4* | 30/30 | I and II pooled | 5 |
| *Pt-fkh* | 30/30 | I and II pooled | 5 |
| *Pt-hh* | 30/30 | I and II pooled | 5 |
|  |  |  |  |
| **Figure 4** | | | |
| *Pt-cad* | 20/20 | I and II pooled | Late 5 |
|  | 20/20 |  | 9 |
| *Pt-Dl* | 17/17 | I and II pooled | Late 5 |
|  | 14/14 |  | 9 |
| *Pt-en* | 10/10 | I and II pooled | 9 |
| *Pt-h* | 15/15 | I and II pooled | Late 5 |
|  | 15/15 |  | 9 |
| *Pt-hh* | 8/8 | I and II pooled |  |
| *Pt-Wnt8* | 11/11 | I and II pooled | Late 5 |
|  | 17/17 |  | 9 |
|  |  |  |  |
| **Figure 5** | | | |
| *Dl* RNAi | 37/37 | Mild and Strong | 7 |
|  | 22/22 |  | 9 |
| *Wnt8* RNAi | 40/40 | Mild and Strong | 7 |
|  | 16/16 |  | 9 |
|  |  |  |  |
| **Figure 2-figure supplement 2** | | | |
| *Pt-Dfd-A* | 9/9 | I and II pooled | 9 |
| *Pt-lab* | 10/10 | I and II pooled | 9 |
| *Pt-twi* | 14/14 | I and II pooled | 9 |
|  |  |  |  |
| **Figure 2-figure supplement 4** | | | |
| Cleaved Caspase-3 | 15/15 | I and II pooled | 5 |
|  | 15/15 | I and II pooled | 9 |
| Phosphohistone H3 | 15/15 | I and II pooled | 5 |
|  | 15/15 | I and II pooled | 9 |
