# Supplementary material for: A SoxB gene acts as an anterior gap gene and regulates posterior segment addition in a spider
Source: eLife. 2018 Aug 21;7:e37567. doi: 10.7554/eLife.37567 (PMC6167052; doi:10.7554/eLife.37567)
Supplement: Supplementary file 3. [file elife-37567-supp3.docx]

| **Oligonucleotide name** | **Sequence (5’ to 3’)** |
| --- | --- |
| **In situ Hybridization probes - Cloning** | |
| Caudal_fw | CCCATGCGGAGTTATGGACA |
| Caudal_rv | GTCCTGGTTCTGCCTGGATT |
| Dfd_A_fw | CCCCTGTAAGTTATGGCCC |
| Dfd_A_rv | AGCACTGGGTTGCTGTTTCT |
| Dpp_fw | ATGCGCCAGCGCATTTGGGCT |
| Dpp_rv | ACGGCAACCACATCCTTCAACAAC |
| Delta_fw | CTGTCGTTTGGGTTGGCAAG |
| Delta_rv | CCCCATTGAGGCATGGTTCT |
| Engrailed_fw | ATGATACCAATGAGAACTCGA |
| Engrailed_rv | CCATTAATTGCAATGCCAGT |
| Ets4_fw | AGGTCCACCTCCCTATGT |
| Ets4_rv | ACGCTCAACGTCACAGGA |
| Fkh_fw | CATGCCCATGTCCCTCAAC |
| Fkh_rv | AAGCGTTTTTGGCGCCTTAG |
| Hairy_fw | AAATACGGCCACAGTCAGGG |
| Hairy_rv | ATCCGAGCTTATGCTCACCG |
| Hedgehog_fw | GTGCCTGGCCGCATTAGTG |
| Hedgehog_rv | TGAGTCACCATCGAAACATC |
| Labial_fw | GGACAACTACGTGCAGGACA |
| Labial_rv | AGCTGAAACAGACGCTCCTC |
| Sox21b-1_fw | ATGCAAGCTCCGCAAATCGTACAAAA |
| Sox21b-1_rv | TTACATCTGTAATGGCATGCCACG |
| Twist_fw | ACGTTAGGACGAATCCACTG |
| Twist_rv | CTGGGCTCTCTGAACCTG |
| Wnt8_fw | CTATGCAGACAGCGTTGCTATTG |
| Wnt8_rv | GGTGAAATTTCATTGTAGATTAGCTGG |
| **dsRNA synthesis** | |
| Dl_dsRNA_fw | TAATACGACTCACTATAGGATGTAAGCGAGTTCTGGACTCAAGACA |
| Dl_dsRNA_rv | TAATACGACTCACTATAGGCACGTTCCTCCATTAGAGCACGGCTTG |
| GFP_dsRNA_fw | TAATACGACTCACTATAGGCGTGTCCGGCGAGGGCGAGGG |
| GFP_dsRNA_rv | TAATACGACTCACTATAGGAGGACCATGTGATCGCGCT |
| Sox21b1_dsRNA_F1_fw | TAATACGACTCACTATAGGATGCAAGCTCCGCAAATCGTAC |
| Sox21b1_dsRNA_F1_rv | TAATACGACTCACTATAGGAGAAGAGGCAGGATAGCCGC |
| Sox21b1_dsRNA_F2_fw | TAATACGACTCACTATAGGTCAAGTGTCTGGATCAGCAGC |
| Sox21b1_dsRNA_F2_fw | TAATACGACTCACTATAGGTTACATCTGTAATGGCATGCCAC |
| Wnt8_dsRNA_fw | TAATACGACTCACTATAGGCTATGCAGACAGCGTTGCTATTG |
| Wnt8_dsRNA_rv | TAATACGACTCACTATAGGGGTGAAATTTCATTGTAGATTAGCTGG |
