# Supplementary material for: A SoxB gene acts as an anterior gap gene and regulates posterior segment addition in a spider
Source: eLife. 2018 Aug 21;7:e37567. doi: 10.7554/eLife.37567 (PMC6167052; doi:10.7554/eLife.37567)
Supplement: Transparent reporting form [file elife-37567-transrepform.docx]

***eLife’s* transparent reporting form**

We encourage authors to provide detailed information *within their submission* to facilitate the interpretation and replication of experiments. Authors can upload supporting documentation to indicate the use of appropriate reporting guidelines for health-related research (see [EQUATOR Network](http://www.equator-network.org/%20)), life science research (see the [BioSharing Information Resource](https://biosharing.org/)), or the [ARRIVE guidelines](http://www.plosbiology.org/article/info:doi/10.1371/journal.pbio.1000412) for reporting work involving animal research. Where applicable, authors should refer to any relevant reporting standards documents in this form.

If you have any questions, please consult our Journal Policies and/or contact us: [editorial@elifesciences.org](mailto:editorial@elifesciences.org).

**Sample-size estimation**

- You should state whether an appropriate sample size was computed when the study was being designed
- You should state the statistical method of sample size computation and any required assumptions
- If no explicit power analysis was used, you should describe how you decided what sample (replicate) size (number) to use

Please outline where this information can be found within the submission (e.g., sections or figure legends), or explain why this information doesn’t apply to your submission:

We planned our knockdown experiment with the injection of two non-overlapping fragments of the Sox21b-1 gene from the spider. For each cocoon of each spider, we collected embryos at two different time-points (formation of the cumulus at germ disc stage 5 – and when the ventral neuroectoderm already is differentiated in neural precursors) and pooled 30 embryos for phenotypical analysis. The sample size for the gene expression is always stated either in the text or in the figure legend for each gene. As in previous studies we assumed that *n* of a minimum of 10 embryos pooled from different cocoons/spiders were enough for the gene expression analysis.

**Replicates**

- You should report how often each experiment was performed
- You should include a definition of biological versus technical replication
- The data obtained should be provided and sufficient information should be provided to indicate the number of independent biological and/or technical replicates
- If you encountered any outliers, you should describe how these were handled
- Criteria for exclusion/inclusion of data should be clearly stated
- High-throughput sequence data should be uploaded before submission, with a private link for reviewers provided (these are available from both GEO and ArrayExpress)

Please outline where this information can be found within the submission (e.g., sections or figure legends), or explain why this information doesn’t apply to your submission:

The double-stranded RNA injections were performed in seven spiders per fragment (total n = 14 for Sox21b-1) and n = 2 for the GFP controls. Therefore, gene expression in knockdown embryos was performed in seven biological replicates and two technical replicates, and two biological replicates and two replicates for control embryos. All of the experimental procedures were performed for the same time length of time (e.g. embryo fixation, chromogenic reaction, DAPI staining).

**Statistical reporting**

- Statistical analysis methods should be described and justified
- Raw data should be presented in figures whenever informative to do so (typically when N per group is less than 10)
- For each experiment, you should identify the statistical tests used, exact values of N, definitions of center, methods of multiple test correction, and dispersion and precision measures (e.g., mean, median, SD, SEM, confidence intervals; and, for the major substantive results, a measure of effect size (e.g., Pearson's r, Cohen's d)
- Report exact p-values wherever possible alongside the summary statistics and 95% confidence intervals. These should be reported for all key questions and not only when the p-value is less than 0.05.

Please outline where this information can be found within the submission (e.g., sections or figure legends), or explain why this information doesn’t apply to your submission:

The percentage of phenotypical appearance was calculated for each group and the raw numbers are stated in supplementary table 2. The sample sizes of embryos for gene expression analysis are provided in the text and/or figure legends.

(For large datasets, or papers with a very large number of statistical tests, you may upload a single table file with tests, Ns, etc., with reference to sections in the manuscript.)

**Group allocation**

- Indicate how samples were allocated into experimental groups (in the case of clinical studies, please specify allocation to treatment method); if randomization was used, please also state if restricted randomization was applied
- Indicate if masking was used during group allocation, data collection and/or data analysis

Please outline where this information can be found within the submission (e.g., sections or figure legends), or explain why this information doesn’t apply to your submission:

The two groups of embryos were from the parental RNAi knockdown females and embryos from the GFP control injected females.

**Additional data files (“source data”)**

- We encourage you to upload relevant additional data files, such as numerical data that are represented as a graph in a figure, or as a summary table
- Where provided, these should be in the most useful format, and they can be uploaded as “Source data” files linked to a main figure or table
- Include model definition files including the full list of parameters used
- Include code used for data analysis (e.g., R, MatLab)
- Avoid stating that data files are “available upon request”

Please indicate the figures or tables for which source data files have been provided:

Supplementary File 1 shows the percentage of embryos containing the phenotypes for each group.
